# Editorial: Physical AI and robotics – outputs from IS-PAIR 2025 and beyond

**DOI:** 10.3389/frobt.2026.1881555

**Published:** 2026-06-04

**Authors:** Poramate Manoonpong, Shunsuke Shigaki, Ronnapee Chaichaowarat, Mikhail Shamonin

**Affiliations:** 1 Bio-inspired Robotics and Neural Engineering Lab, School of Information Science and Technology, Vidyasirimedhi Institute of Science and Technology, Rayong, Thailand; 2 Embodied AI and Neurorobotics Lab, SDU Biorobotics, The Mærsk Mc-Kinney Møller Institute, University of Southern Denmark, Odense, Denmark; 3 Principles of Informatics Research Division, National Institute of Informatics, Tokyo, Japan; 4 International School of Engineering, Faculty of Engineering, Chulalongkorn University, Bangkok, Thailand; 5 Ostbayerische Technische Hochschule Regensburg, Regensburg, Germany

**Keywords:** artificial intelligence, embodiment, IS-PAIR, morphological computation, neural computation, robotics

Physical Artificial Intelligence (AI) is an emerging interdisciplinary field that not only integrates AI and robotics but also considers dynamic interactions with the environment. It seeks to develop intelligent systems capable of adapting and interacting with complex and dynamic environments. Central to this field is the integration and interaction of three fundamental components: the digital brain, the physical body, and the environment. The “brain” encompasses advanced computational systems, including sensory signal processing, motor control, and learning. These systems process sensory data, translate it into proper motor commands, and even make predictions, active adaptations, and decisions to deal with unexpected conditions. The “physical body” refers to structures, actuation systems, and materials that generate actions and enable physical interaction with the real world. It also generates passive adaptations or preflexes to handle a certain degree of perturbations and automatically stabilize system movements. The environment, in turn, provides challenges and opportunities that drive the system’s adaptability and performance, and can also be exploited to produce complex emergent behaviors.

Drawing inspiration from biological systems, which excel at navigating and adapting to their surroundings, this field aims to create artificial systems that can behave, predict, learn, adapt, and even improve over time. By bridging advances in artificial intelligence, biomechanics, material science, and robotics, Physical AI has the potential to transform industries by enabling robots with advanced computing to operate efficiently in unpredictable and unstructured environments.

This Research Topic, “Physical AI and Robotics,” partnered with the first International Symposium on Physical Artificial Intelligence and Robotics (IS-PAIR) 2025, aims to tackle the challenge of creating intelligent systems capable of functioning in highly dynamic and uncertain environments. The key objectives of this Research Topic are: 1. To strengthen the fundamentals of developing advanced algorithms for real-time decision-making and continuous learning, enabling (physical) systems to adjust their behavior and decisions based on environmental changes. 2. To encourage creativity in designing flexible, durable robotic structures using biomimetic materials that can withstand unpredictable conditions, ensuring consistent performance in diverse settings. 3. To examine the role of creating sensory systems and actuators that allow robots to perceive their environment accurately and respond with adaptive, proactive, precise actions. This Research Topic comprises seven articles, covering a broad spectrum of Physical AI research from material and structural design to control and machine learning theories, as well as the practical application of these results in robot locomotion and manipulation. Among these, one study proposes a novel technique for obtaining the adaptive behavioral data of insects to serve as a resource for future Physical AI development.

In the domain of machine learning for Physical AI, one study (Kobayashi) proposes two novel methods to balance memory consolidation and plasticity (weight adaptation) within continual learning (CL) frameworks. The first method, called A2ER, incorporates three sub-strategies: 1) the adaptation strategy for auto-tuning of the weights and balancing the learning from new and past data, as well as the preservation of past outputs; 2) the block strategy for preventing memory consolidation of incorrect skills; and 3) the correction strategy for updating past outputs to make them consistent with the current situation and enhance plasticity. The second method, called O2S, consists of three other mechanisms: 1) the q-logarithm strategy for facilitating the specification of the balance between consolidation and plasticity; 2) the plural strategy for shifting from high plasticity to consolidated processes; and 3) the omission strategy for filtering out inconsistent past data and preserving important data for long-term memory. These improvements were tested in various benchmark tasks, such as regression, classification, and reinforcement learning problems. Another study (Takahashi et al.) investigates a novel bio-inspired nonlinear update rule for value and policy functions in reinforcement learning (RL). It introduces a theoretical framework with the Weber–Fechner law (WFL), which can utilize the nonlinearity between the degree of updates (perception) and temporal difference (TD) errors (stimulus change). This framework can accelerate the escape from low-reward situations and minimize the impact of punishments during the learning process. Its efficacy was validated and demonstrated using three 3-DOF robotic fingers tasked with learning valve manipulation. In addition to robotic valve manipulation, cloth unfolding and folding represent fundamental challenges within the domain of Physical AI. Recently, several studies have actively tried to address the challenges using deep learning methods. From this perspective, a review (Gu et al.) identifies and summarizes current progress in deep learning-based cloth unfolding and folding. The review covers 41 relevant papers from 2019 to 2024 and investigates various factors influencing manipulation. It describes the remaining challenges, including handling irregular cloth sizes and diverse initial garment states, improving real-world data Research Topic systems, and requiring more realistic cloth simulators, together with the Sim2Real gap. Furthermore, multimodal sensory integration and the emergence of primitive actions must be addressed to enhance overall performance. The review also provides future perspectives for Physical AI in this domain.

To enable machine learning and deep learning systems to function effectively in real-world environments, the appropriate design of the body is indispensable; in this regard, the concept of embodiment (Pfeifer and Bongard, 2006) plays a pivotal role. Conventional machine learning has predominantly evolved within a framework that learns input–output mappings from large-scale datasets. Within this paradigm, intelligence is generally regarded as being realized through internal representations, such as neural networks, while the body is often treated as a fixed condition or a mere output device. However, when considering the behavior of biological organisms in real-world settings, factors such as sensory uncertainty, environmental dynamics, and the structural and material properties of the body play essential roles in the generation of behavior. This crucial insight has long been investigated under the notion of embodiment, and in recent years, Physical AI has gained increasing attention as a framework for constructing AI systems that operate in the real world, grounded in this concept. Purely data-driven approaches exhibit inherent limitations in adaptability and robustness when confronted with novel environments; consequently, Physical AI, which conceptualizes intelligence as emerging from interactions among the body, brain, and environment, has become a focal point of contemporary research.

Within this context, the four original articles featured in this Research Topic (Sekiwa et al.; Ohashi et al.; Wang et al.; Maeta et al.) collectively and complementarily address different layers constituting Physical AI. First, a study employing an insect-tracking robot proposes a system that simultaneously measures olfactory reception (EAG) and behavior in freely moving insects (Sekiwa et al.). This approach elucidates the coupling between sensory inputs and behavior in real environments—previously inaccessible under constrained experimental conditions—and demonstrates that insects modulate their search strategies based not merely on concentration gradients but on the temporal dynamics of odor signals. This work provides empirical data on perception–action loops mediated by the body, thereby constituting the foundational “measurement” layer in Physical AI. Second, a study on biohybrid robots utilized three-dimensional muscle tissues as actuators and systematically investigated how differences in the extracellular matrix (ECM) influence contractile force and response characteristics (Ohashi et al.). The findings reveal that the selection of materials and structural configurations significantly affects not only motor performance but also reproducibility and stability. This implies that intelligent behavior is not determined solely by algorithms but profoundly shaped by the nature of the body itself. In other words, body design is inherently part of intelligence design, underscoring the critical importance of the “body” in Physical AI. Third, from the perspective of control, Wang et al. implemented a “dual-reflex system” in a musculoskeletal robot driven by pneumatic artificial muscles and examined its adaptability to external disturbances. This study exemplifies how complex adaptive behaviors can emerge from the application of simple local rules to a compliant body. Finally, Maeta et al. investigated the generation mechanism of footfall patterns in turning gallops of quadrupeds using a simplified robotic model with decentralized control. They demonstrated that the emergence of an inner-leading limb can be induced solely by trunk roll motion and local load feedback, without reliance on sophisticated central control. These findings highlight that body structure and physical interactions play a decisive role in behavior generation, strongly supporting the significance of embodiment and decentralized control.

In summary, the contributions to this Research Topic emphasize the importance of Physical AI as a holistic framework. This framework not only drives the development of new robotics and machine learning technologies for real-world applications but also facilitates a deeper investigation into the control and learning strategies underlying the adaptive behaviors of biological systems. We hope that these articles will encourage further collaboration across related research fields to advance Physical AI for industrial and societal benefits.

## References

[B1] Pfeifer R. Bongard J. (2006). How the Body Shapes the Way We Think: A New View of Intelligence. MIT Press.

